# Challenging pharmacotherapy management of a psychotic disorder due to a delicate pharmacogenetic profile and drug-drug interactions: a case report and literature review

**DOI:** 10.3325/cmj.2024.65.383

**Published:** 2024-08

**Authors:** Andrej Belančić, Aristea Pavešić Radonja, Lana Ganoci, Dinko Vitezić, Nada Božina

**Affiliations:** 1 Department of Clinical Pharmacology, Clinical Hospital Centre Rijeka, Rijeka, Croatia; 2 Department of Basic and Clinical Pharmacology with Toxicology, University of Rijeka, Faculty of Medicine, Rijeka, Croatia; 3 Psychiatry Clinic, Clinical Hospital Centre Rijeka, Rijeka, Croatia; 4 Department of Psychiatry and Psychological Medicine, University of Rijeka, Faculty of Medicine, Rijeka, Croatia; 5 Division of Pharmacogenomics and Therapy Individualization, Department of Laboratory Diagnostics, University Hospital Centre Zagreb, Zagreb, Croatia; 6 Department of Pharmacology, School of Medicine, University of Zagreb, Zagreb, Croatia; The last two authors equally contributed.

## Abstract

This report presents challenging psychopharmacotherapy management of a psychotic disorder in a patient with a delicate pharmacogenetic profile and drug-drug interactions. A 31-year old woman diagnosed with schizophrenia in 2017 was referred by her psychiatrist to a clinical pharmacologist for interpretation of a pharmacogenetic test and advice regarding optimal psychopharmacotherapy. In spite of adherence to aripiprazole, olanzapine, risperidone, and levomepromazine, and rational anxiolytic therapy, she still experienced anxiety, anhedonia, loss of appetite, sleeping problems, and auditory hallucinations with commands to harm herself. Due to a lack of alternative therapeutic steps, low aripiprazole serum concentration, and a lack of explanation for pharmacotherapy unresponsiveness, pharmacogenetic testing was performed. The patient was defined as CYP2D6 *1/*1, CYP1A2 *1F/*1F, CYP3A4 *1/*1B, CYP3A5 *1/*3, and having increased activity of the enzymes UGT1A4 and UGT2B7, intermediate activity of ABCB1 transporter, and low activity of COMT. Carbamazepine was discontinued, aripiprazole was increased to a maximum of 30 mg/day orally with long-acting injection (400 mg monthly), and olanzapine was increased to a daily dose of 35 mg orally. These changes led to an optimal therapeutic drug concentrations and improved clinical status. At the last follow-up, the patient was without severe auditory hallucinations, became more engaged in daily life, had more interaction with others, had found a job, and even had started an emotional relationship. In psychiatry, pharmacogenetic testing is an important tool for guiding pharmacological therapy, particularly in patients with an unsatisfactory clinical response and a lack of alternative therapeutic steps for pharmacotherapy unresponsiveness.

Personalized medicine is a steadily growing field whose value lies in the prediction of individual responses to drugs regarding effectiveness, safety profile, and pharmacokinetics. Yet, there are still disagreements on its therapeutic applicability. As 25%-50% of people do not respond normally to drug treatment and standard dosage, personalized medicine calls for the development of resources for clinicians and pharmacogenetic (PGx) dosing guidelines for medicines. This is especially important since about 90% of patients undergoing PGx testing are likely to achieve a clinically actionable result (1).

PGx may be particularly important in mental health. The Food and Drug Administration (FDA) identified approximately 20% of the 121 pharmacogenetics markers as useful for clinical practice concerning psychiatric medicines (2). Implementation of PGx helps the selected patients and their referring clinicians to attain therapeutic drug concentrations more quickly, which ensures a safe, efficacious, proactive, and cost-effective approach (3,4). In psychiatry, a prompt clinical response is highly preferred since those patients in acute states are mostly uncooperative and incapable of everyday functioning on their own. Most antipsychotics/antidepressants are metabolized by CYP3A4, CYP2D6, and CYP2C19 enzymes in the liver (5). Genetic variants that affect the functioning of these enzymes are widespread (62% of people across the globe), and are strongly interconnected with enzymatic activity. Therefore, *CYP2D6* and *CYP2C19* have been the focus of Clinical Pharmacogenetics Implementation Consortium (CPIC) guidelines on the clinical use of PGx in psychiatry (6-8).

Antipsychotic medications *per se* do not cure schizophrenia, but they can alleviate its symptoms and improve the patient’s quality of life. Before optimal antipsychotic medicines and doses are found, many patients frequently experience a trial-and-error phase marked by poorly managed symptoms and/or severe drug reactions (9,10). Antipsychotic medication response varies substantially between patients; hence, multiple trials are often required to identify the medication that is most effective and tolerable for each individual. Thus, PGx may serve as a valuable tool, especially in treatment-resistant schizophrenia (present in up to 30% of people with schizophrenia) or in cases complicated by unexplained adverse drug reactions (ADRs) (10-12).

As various prescribed or over-the-counter medicines, or dietary sources, and their interactions may act as inhibitors or inducers of cytochrome P450 gene, in psychotropic medication management a thorough medical history-taking is necessary. In the upcoming years, patients’ pharmacogenetics data are going to become more widely integrated into clinical practice and electronic health. Also, psychiatric-pharmacogeneticist-clinical pharmacologist multidisciplinarity is going to become even more essential (1), particularly in psychiatry, where patients are frequently managed by numerous medicines.

Here, we report on a challenging psychopharmacotherapy management of a psychotic disorder in a patient who, in spite of adherence to high doses of four concomitant antipsychotics, at first did not achieve clinical response (in terms of auditory hallucinations control) due to pharmacogenetic predisposition and class-D drug-drug interactions. This article highlights the importance of pharmacogenetics and multidisciplinarity in psychiatry.

## Case description

A 31-year old female patient, diagnosed with schizophrenia in 2017, was referred by her psychiatrist to a clinical pharmacologist for interpretation of a pharmacogenetic test and advice regarding optimal psychopharmacotherapy. At the outpatient visit (November 2021), her chronic pharmacotherapy consisted of a long-acting injection of aripiprazole (400 mg i.m. monthly), aripiprazole (10 mg/day orally), olanzapine (10 + 0 + 15 mg/day orally), risperidone (2 + 0 + 2 mg/day orally), levomepromazine (25 mg/day per need orally), carbamazepine (400 mg in the morning orally), oxazepam (15-15-30 mg/day orally), alprazolam (0.5 mg in the morning orally), biperiden (per need orally), bisoprolol (5 mg in the morning orally), and metoclopramide (10 mg in the morning orally). Before this antipsychotic regimen (after non-response to two antipsychotics), an attempt with clozapine was also made; however, it had to be terminated due to side effects (predominantly tachycardia).

In spite of adherence to four antipsychotics and anxiolytic medications, she still experienced anxiety, anhedonia, loss of appetite, sleep problems, and auditory hallucinations with imperative commands to harm herself. Due to a lack of alternative therapeutic steps, low aripiprazole serum concentrations (190 nmol/L; reference range: 334-1115 nmol/L), and a lack of explanations for pharmacotherapy unresponsiveness, pharmacogenetic testing was performed. We tested polymorphisms in the genes of metabolic enzymes and drug transporters involved in the metabolism of drugs listed in the patient’s pharmacotherapy. They were primarily selected according to pharmacogenetic guidelines. Also, they were selected based on the literature data for variants that may have functionally significant effects on enzyme or transporter function, or had been investigated for association with interindividual variability in drug response, drug pharmacokinetics, or adverse drug reactions. Pharmacogenetic polymorphisms *ABCB1 c.3435C*>*T* (rs1045642), *ABCG2 c.421C*>*A* (rs2231142), *COMT c.472G>A* (rs4680), CYP1A2*1F (rs762551), CYP2C9*2 (rs1799853), CYP2C9*3 (rs1057910), CYP2C19*2 (rs4244285), CYP2C19*17 (rs12248560), CYP2D6*3 (rs35742686), CYP2D6*4 (rs3892097), CYP2D6*6 (rs5030655), CYP2D6*9 (rs5030656), CYP2D6*10 (rs1065852), CYP2D6 *41 (rs28371725), CYP3A4*1B (rs2740574), CYP3A4*22 (rs35599367), CYP3A5*3 (rs776746), UGT1A4*2 (rs6755571), and *UGT2B7 c.-161C>T* (rs7668258) were genotyped with TaqMan SNP Genotyping Assays (Applied Biosystems, Foster City, CA, USA) on a 7500 Real-Time PCR System (Applied Biosystems), according to the manufacturer’s instructions. UGT1A4*3 (rs2011425) was genotyped with Custom TaqMan SNP Genotyping Assay (Applied Biosystems), as previously published (13). CYP2D6*5 gene deletion and *CYP2D6* duplications were genotyped by long-range polymerase chain reaction (14), and confirmed with TaqMan CNV assays for exon 9 (Hs00010001_cn), intron 2 (Hs04083572_cn), and intron 6 (Hs04502391_cn) (Applied Biosystems), on a 7500 Real-Time PCR System (Applied Biosystems), according to the manufacturer’s instructions.

Therapeutic drug monitoring (TDM) analysis of aripiprazole, carbamazepine, olanzapine, and risperidone was performed by high-performance liquid chromatography (Shimadzu Corporation, Kyoto, Japan). Genotyping and TDM methods were implemented and validated for routine testing in an accredited laboratory at the University Hospital Centre Zagreb. Genotyping results and their corresponding phenotypes are shown in Table 1.

**Table 1:** Pharmacogenetic analysis report and pharmacotherapy

Gene - allele	Genotype	Phenotype	Substrate	Inducer	Inhibitor
Enzymes					
COMT c.472G>A	A/A	poor enzyme activity			
CYP1A2 *1F	*1F/*1F	increased inducibility, which can result in ultrarapid metabolism in the presence of inducer	carbamazepine metoclopramide olanzapine	carbamazepine	
CYP2C9 *2, *3	*1/*1	normal metabolizer	alprazolam	carbamazepine	
CYP2C19 *2, *17	*1/*1	normal metabolizer		carbamazepine	
CYP2D6 xN, *3, *4, *5, *6, *9, *10, *41	*1/*1	normal metabolizer	aripiprazole fluphenazine levomepromazine metoclopramide olanzapine risperidone		fluphenazine metoclopramide levomepromazine
CYP3A4 *1B, *22	*1/*1B	normal metabolizer	alprazolam aripiprazole bisoprolol carbamazepine metoclopramide olanzapine risperidone	carbamazepine	
CYP3A5 *3	*1/*3	CYP3A5 expressor (intermediate metabolizer)	alprazolam aripiprazole carbamazepine olanzapine risperidone	carbamazepine	
UGT1A4 *2, *3	*3/*3	high enzyme activity	olanzapine	carbamazepine	
UGT2B7 c.-161C>T	T/T	substrate specific enzyme activity	carbamazepine	carbamazepine	
Transporters					
ABCB1 c.3435C>T	C/T	intermediate function	bisoprolol metoclopramide olanzapine risperidone	carbamazepine	aripiprazole bisoprolol
ABCG2 c.421C>A	C/C	normal function	risperidone		aripiprazole

**Figure 1:**
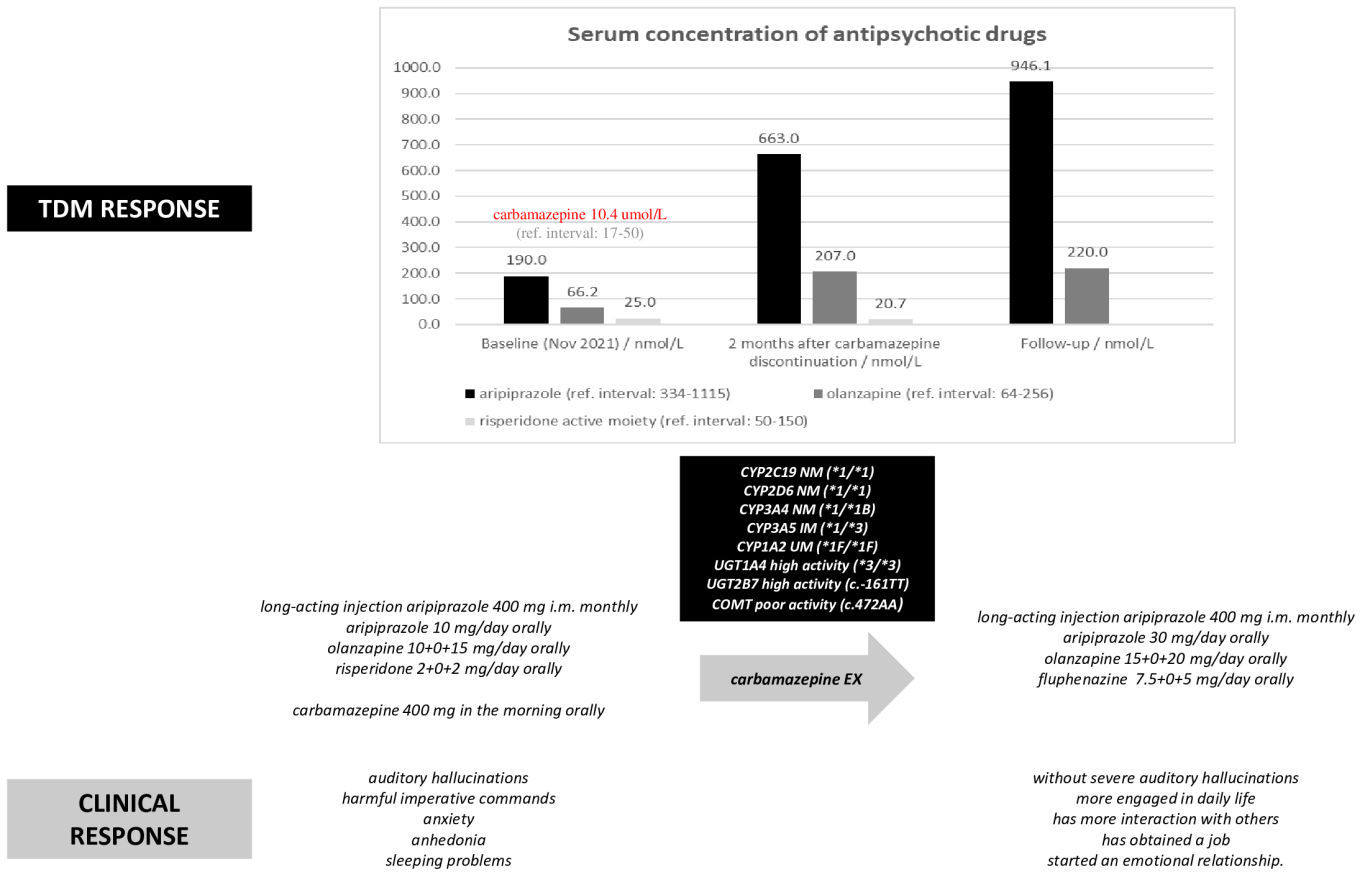
Therapeutic drug monitoring (TDM) and clinical responses to pharmacogenetic-guided pharmacotherapy management.

The patient was defined as normal CYP2C19 (CYP2C19 *1/*1), CYP2D6 (CYP2D6 *1/*1), and CYP3A4 (CYP3A4 *1/*1B) metabolizer; CYP3A5 (CYP3A5 *1/*3) exspressor; and ultrarapid CYP1A2 (CYP1A2 *1F/*1F) metabolizer. She had also high activity of the enzyme UGT1A4 (UGT1A4 *3/*3) and of UGT2B7 for some substrates (*UGT1A4 c.-161TT),* intermediate activity of the ABCB1 transporter *ABCB1 c.3435CT*, and poor activity of COMT *(COMT c.472AA*). Based on these results, carbamazepine (strong CYP3A4 inducer) and risperidone were discontinued, and aripiprazole (substrate of CYP2D6 and CYP3A4) was increased to a maximum of 30 mg/day orally (with a long-acting injection of aripiprazole 400 mg monthly), and olanzapine was increased to a daily dose of 35 mg orally (75% higher than the approved daily dose). This regimen finally resulted in optimal therapeutic drug concentrations and an improved clinical status (Figure 1). At her last ambulatory check-up (two years after carbamazepine discontinuation), the patient was without severe auditory hallucinations, had become more engaged in daily life, had more interaction with others, had obtained a job, and even had started an emotional relationship. The antipsychotic therapy consisted of aripiprazole long-acting injection (400 mg i.m. monthly), aripiprazole (30 mg/day orally), olanzapine (15 + 0 + 20 mg/day orally), and fluphenazine (7.5 + 0 + 5 mg/day orally). The most recent Positive and Negative Syndrome Scale score was 67, specifically comprising positive 17/negative 17/cognitive or general psychopathology 33; while she was deemed moderately ill on the Clinical Global Impression scale (15).

## Discussion

Here, we report on a patient with schizophrenia who did not adequately respond to applied pharmacotherapy and experienced side effects (Figure 1). In order to determine the influence of gene polymorphisms relevant to the metabolism and transport of the applied drugs, a pharmacogenetic analysis was performed.

The patient’s treatment was not effective despite the use of several antipsychotics. Among the possible causes are lower-than-expected concentrations of the administered drugs (primarily aripiprazole, olanzapine, and risperidone) due to, at least in part, pharmacogenetic predisposition and drug-drug-gene interactions. Pharmacogenetic analysis showed that the patient had normal or increased activity of phase-I (CYPs) and phase-II metabolic enzymes (UGTs), intermediate activity of the ABCB1 transporter, and low activity of COMT. In addition, her therapy included carbamazepine, a strong inducer of several enzymes and transporters. All of these factors contributed to treatment failure. The patient’s condition improved after discontinuing carbamazepine, replacing risperidone, and adjusting the dose according to the available guidelines and relevant literature.

Several points need to be discussed for a better understanding of this case. By translating the analyzed genotypes into phenotypes, we tried to clarify the background for treatment failure. Treatment personalization for patients experiencing treatment failure or ADRs requires an accurate estimation of enzyme activity.

### Aripiprazole

The main enzymes involved in the metabolism of aripiprazole are CYP2D6 and CYP3A4, and their gene variants can modulate its pharmacokinetics and eventually dosing (16,17). Aripiprazole’s main metabolite, dehydro-aripiprazole (D-ARI), has similar properties as the parent drug but, according to some authors, even more potent effects (18,19).

The DPWG’s guidelines listed relevant interactions of the *CYP2D6, CYP3A4,* and *CYP1A2* genes and antipsychotic drugs (20). Medications requiring dose adjustment in patents with the *CYP2D6* genotype included aripiprazole and risperidone, among others (brexpiprazole, haloperidol, pimozide, and zuclopenthixol).

The FDA and DPWG issued recommendations to reduce the doses of aripiprazole and risperidone in CYP2D6 poor metabolizers. In CYP2D6 ultrarapid metabolizers, better options are treatment with an alternative drug instead of risperidone or dose titration by measuring concentrations of the active metabolite paliperidone (9-hydroxyrisperidone) (20-24). Additionally, an adjustment and re-evaluation of the applied dose are recommended in cases of polytherapy with interacting drugs (22,23,25-27). For example, in cases of concomitant administration of aripiprazole and CYP3A4 inhibitors, the dose should be a quarter of the normal dose. DPWG also pointed out that pre-emptive testing for *CYP2D6* is not necessary for all patients, but the decision should be made on an individual basis.

Pharmacogenetic analysis showed that our patient was a CYP2D6 normal metabolizer, and this genotype did not require dose adjustment. However, a gene variant that may contribute to faster metabolism via CYP2D6 (rs5758550) was not included in our diagnostic panel (28,29). For a better prediction of enhanced CYP2D6 enzyme activity, variants rs5758550 and *2 should be included in the test panel (29). Haplotype-based (rs5758550 and rs16947) prediction of CYP2D6 activity was more accurate than the prediction based on variant CYP2D6*2A alone. However, according to Dinh et al (28), the “enhancer” single nucleotide polymorphism (SNP) played a modest role in the prediction of the CYP2D6 phenotype. It had a more pronounced effect on atomoxetine than on dextromethorphan, which suggested potential substrate dependency (28). According to *in vitro* data, CYP2D6*2 alleles and the “enhancer” SNP, taken together, predispose for a modestly higher formation of metabolites (28). This observation was not confirmed *in vivo*, and no enhanced effect was observed for the combination of CYP2D6*1 and the enhancer SNP (28).

Another enzyme significant for the metabolism of aripiprazole is CYP3A4. CYP3A4, in addition to CYP2D6, is included in the dehydrogenation pathway involved in the formation of metabolites. The relevance of the gene variants coding for metabolic enzymes (CYP2D6, CYP3A4, and CYP3A5) and the ABCB1 drug transporter for aripiprazole pharmacokinetics was confirmed in healthy volunteers (30).

The human CYP3A enzyme subfamily is involved in the metabolism of approximately 50% of drugs used in clinical practice. CYP3A4 is expressed in the liver and intestine, while CYP3A5 is found dominantly in extrahepatic tissues. Both enzymes often have the same substrates and exhibit significant variability in their activity. Moreover, variability in populations is very high (31). Their drug substrates also include some psychotropic drugs. Because they have similar structure, function, and substrates, the relative involvement of CYP3A4 and CYP3A5 in drug biotransformation is hard to distinguish (32).

Since CYP3A4 is highly inducible, the relevance of genetic analysis is controversial. Environmental factors such as food, smoking, and other concurrently administered drugs, such as azole compounds, antibiotics, antiepileptics, and cyclosporine, have a stronger role than genetic polymorphisms. For example, citrus fruits, especially grapefruit juice, significantly inhibit CYP3A4 already at the intestinal level, but also on the drug transporter, P-glycoprotein, all of which can increase the bioavailability of drug-substrates.

An active CYP3A5 enzyme is present in only 10% of white people. People who have at least one active CYP3A5*1 allele are called expressors. Most of the population are nonexpressors due to a frequent mutation within intron 3 (rs776746) that predisposes to a splicing defect and formation of a protein with no enzyme activity. The prevalence of nonexpressors differs across populations (33). Guidelines on dosing according to *CYP3A5* have been published only for tacrolimus (34,35).

Data on the influence of *CYP3A4* gene polymorphisms on the expression and function of the enzyme are inconsistent. Some studies associate the variant CYP3A4*1B (–392A>G, rs2740574) in the promoter region with increased CYP3A4 activity, which influences substrate drug metabolism. Others argue that increased CYP3A activity is the result of the presence of the CYP3A5*1 functional allele with which CYP3A4*1B is in strong linkage disequilibrium (LD). It is complex to distinguish which variant is more important for accelerated metabolism (36). A meta-analysis (37) found that kidney transplant patients with the CYP3A4 *1/*1 genotype needed lower doses of tacrolimus than carriers of CYP3A4*1B. Also, tacrolimus trough concentrations were higher in CYP3A4*1/*1 genotype carriers compared with CYP3A4*1B carriers. New *CYP3A4* and *CYP3A5* genotyping recommendations consider CYP3A4*1B to be associated with normal enzyme activity (38).

The most studied *CYP3A4* polymorphisms are *20 (loss-of-function) and *22 (reduced function) alleles, which could predispose to higher concentrations of drugs (39,40). Carriage of CYP3A4*20 can lead to a higher AUC0-t of aripiprazole and a lower AUC0-t of D-ARI, which can result in increased aripiprazole concentrations (30). CYP3A4*22 affects the metabolism of quetiapine (41), but a correlation with the pharmacokinetic parameters of aripiprazole or D-ARI was not confirmed (30).

CYP3A5 has a less significant role in aripiprazole metabolism than CYP2D6 (30). Nonexpressors with genotype CYP3A5 *3/*3 have higher levels of aripiprazole than expressors, such as our patient, who had lower concentrations of substrate drugs.

The published results regarding CYP3A5 are ambiguous. One study in Japanese patients revealed no effect of CYP3A5 variability on the plasma levels of either aripiprazole or its active metabolite (42). Another study found lower values for D-ARI/aripiprazole ratio in nonexpressors (*3/*3) than in expressors (*1/*1 and *1/*3) (30). Besides, the authors also reported the relevance of the *1 variant for the development of ADRs, especially nausea and vomiting, which were conditional on aripiprazole AUC. Dizziness was more common in female carriers of the CYP3A5 *1/*1 genotype (30). The study also confirmed the relevance of CYP2D6 phenotype, *ABCB1 C1236T* gene variants, and sex for the pharmacokinetics of aripiprazole, and *CYP2D6* and *C1236T* for D-ARI. The CYP2D6 phenotype and CYP3A5*3 gene variant were also found to modulate the D-ARI/aripiprazole ratio. The authors found sex, aripiprazole exposure, CYP3A5*3, and the CYP2D6 phenotype relevant for the development of ADRs (30).

*CYP3A* (*3A4/5*) gene variants may also have contributed to low drug concentrations of aripiprazole in our patient. Variants that increase CYP3A4/5/7 expression have been recently reported by the Collins group (43). According to their study (43), the expression of CYP3A4, CYP3A5, and CYP3A7 is controlled by a distal regulatory region (DRR). In addition, using reporter gene assays, variants rs115025140 and rs776744/rs746742 were associated with altered DRR transcriptional activity and higher expression of CYP3A4 and CYP3A5, respectively (43). In liver samples, rs115025140 variant was found to be associated with higher expression of CYP3A4 mRNA and protein, and rs776744/rs776742 with higher expression of CYP3A5 mRNA (43). Since rs115025140 is found only in the population of African descent, African American carriers of this variant who take statin therapy may have lower efficiency in reducing lipids, possibly due to accelerated metabolism of statins via the CYP3A4 enzyme (43). Variants rs776744 and rs776742 were also associated with reduced exposure to tacrolimus in Chinese patients (43). These data could be clinically relevant in predicting CYP3A4 and CYP3A5 activity in specific populations carrying these variants.

The variability in drug exposure may also be attributed to variability in drug transporter activity. Both aripiprazole and D-ARI seem to be substrates of the ATP-binding cassette sub-family B member 1 transporter (ABCB1), called P-glycoprotein (P-gp) (44). P-gp is an efflux transporter involved in the intestinal absorption, transfer across the blood-brain barrier, and excretion of several antipsychotics, modulating their disposition and bioavailability in the brain (14,30,45,46).

According to some studies, P-gp expression and function and, consequently, drug concentrations are modulated by the gene variant *ABCB1 c.3435T>C* (rs1045642). T/T genotype carriers having less duodenal expression of P-gp could have higher levels of drugs (47). But there are also opposite claims in the literature (48,49). Besides *3435T>C* SNP, two other *ABCB1* gene variants, *c.2677T>G/A* (rs2032582) and *c.1236C>T* (rs1128503), being in strong LD, have been related to aripiprazole pharmacokinetics in different studies. However, the results are still controversial and there are no dosage guidelines. For aripiprazole, carriers of variant *ABCB1* alleles *2677T>G/A* (rs2032582) and *3435T>C* (rs1045642) had lower plasma concentrations (50). The pharmacokinetic parameters of aripiprazole and D-ARI were affected by the *ABCB 1236C>T* variants. Higher values for aripiprazole clearance, AUC0-t, and maximum serum concentration for D-ARI, were observed in *C/C* compared with *T/T* carriers (30). Others reported no differences between *ABCB1* variants (42,51). P-gp shares many substrates with CYP3A enzymes and, like them, can be influenced by drug inducers or inhibitors. Often, compounds that induce or inhibit CYP3A4 have the same effect on P-gp, additionally modulating drug bioavailability and effectiveness (52).

As our patient’s genotype indicated intermediate P-gp activity, we cannot conclude on the influence of the *ABCB1* polymorphism. However, P-gp induction by carbamazepine (resulting in a stronger efflux pump) could be relevant for a lower absorption of aripiprazole, thereby contributing to its lower concentration.

### Risperidone

DPWG recommended risperidone dose adjustment related to the *CYP2D6* genotype (20,21,24). A reduced dose is proposed for CYP2D6 poor metabolizers. For CYP2D6 ultrarapid metabolizers, it is recommended to choose an alternative drug or to titrate the dose in relation to the concentrations of the metabolite paliperidone (9-hydroxyrisperidone) (20,24). Additionally, the dose is to be adjusted and re-evaluated when the patient's therapy includes concomitant administration of CYP3A4 inhibitors and/or inducers or CYP2D6 inhibitors (25-27).

In our previous research, CYP2D6 normal/ultrarapid metabolizers (vs other) had lower exposure to risperidone, and *ABCB1* “wild type” allele carriers had 4-fold lower odds of response (14). *ABCB1* variants, *c.1236C>T* (rs1128503) and *c.2677G>T/A* (rs2032582), have been associated with the effectiveness of several antipsychotics such as risperidone, clozapine, and haloperidol (53).

In our patient, CYP3A4*1B/3A5*1 variants could have also contributed to the accelerated metabolism of risperidone. Although carbamazepine was discontinued, the concentrations of risperidone and its active metabolite were still very low. Therefore, we considered that the influence of genetic predisposition for increased metabolism by CYP3A was relevant, and risperidone was switched to fluphenazine.

Literature data for CYP3A5 variants are controversial. While one study showed that *CYP3A5* *3 carriers had higher levels of risperidone, 9-hydroxyrisperidone, and active moiety than CYP3A5*1 carriers (54), two other studies did not confirm this for the CYP3A phenotype (14,55), possibly due to the small sample size.

We also analyzed the *COMT* polymorphism, since some *COMT* variants influence antipsychotic response. The rs9606186 variant was found to affect the efficacy of risperidone in male patients (56), while for rs165599, the association with risperidone’s impact on negative symptoms was observed (57). Our patient was homozygous for the variant allele *COMT c.472G>A* (p.Val158Met, rs4680), and variant allele A was found to be associated with a decreased response to risperidone (58).

### Olanzapine

The enzymes involved in phase I of olanzapine metabolism are CYP1A2, CYP2D6, and CYP3A4, and those involved in phase II are glucuronidation enzymes UGT1A4 and UGT2B10 (59,60).

Although relevant for olanzapine biotransformation, many studies have not demonstrated the association of CYP2D6 activity and olanzapine plasma levels (59,61-63). This is probably due to the secondary role of this enzyme in olanzapine biotransformation. Therefore, there are no recommendations for dosing based on the CYP2D6 phenotype. The EMA and FDA recommend that in patients treated with CYP1A2 inhibitors, a lower starting dose is considered (64,65). In cases of concomitant therapy with CYP3A4 and ABCB1 inducers or inhibitors, an appropriate dose for the patient has to be determined (64). Important factors that can modulate the bioavailability and efficacy of CYP1A2 substrates include drug-drug interactions and environmental factors such as smoking and diet (caffeine consumption, cruciferous vegetables, polyamine hydrocarbons from grilled meat) (66). These factors have been shown to induce CYP1A2, while fluoroquinolone antibiotics and oral contraceptives have been shown to reduce it (67).

Among the genetic variants of CYP1A2, CYP1A2*1F (*c. −163C>A*, rs762551) SNP is the most well-studied. According to the Pharmacogenomics Knowledgebase, the CYP1A2*1F variant has been associated with an altered phenotype (68). Because of increased induction of expression, it contributes to an accelerated metabolism of substrate drugs (ultrarapid metabolism). However, this effect is conditional on the concomitant use of an inducer such as smoking or heavy coffee consumption (69). In white smokers, carriage of the *CYP1A2 -163 AA* genotyp*e* predisposed for increased caffeine metabolism, while this effect was not observed in non-smokers (70). Drugs can also act as CYP1A2 inducers. Omeprazole taken by non-smoking healthy carriers of CYP1A2 *1F/*1F genotype compared with those with CYP1A2 *1C/*1F genotype increased the induction of caffeine metabolism (71). The CYP1A2*1F variant has been associated with increased enzyme inducibility and consequently lower agomelatine (*CYP1A2* substrate) exposure (72). The latter study also observed a significant inter-racial difference in the frequency of *CYP1A2* gene variants (72).

In our case, the use of carbamazepine as an inducer could have turned our patient, an *1F/*1F carrier, into an ultrarapid metabolizer. In several studies, the CYP1A2*1F variant was associated with lower olanzapine concentrations (59,61,73), which implies that the presence of the CYP1A2*1F allele enhances the inducibility of the enzyme when concomitant therapy with inducers is used. Significantly lower olanzapine concentrations and, consequently, the ineffectiveness of olanzapine therapy, were observed in *1F carriers compared with *1/*1 carriers. DPWG and CPIC recommend no dose adjustment for olanzapine and *CYP1A2* genotypes.

There are no data on the accelerated metabolism of antipsychotics associated with the variants CYP3A4*1B and CYP3A5*1, but some conflicting data are provided for olanzapine and non-function allele CYP3A5*3 (61,74). As discussed earlier for aripiprazole, new SNP variants in the DRR that alter transcriptional activity (not tested in this patient) could have contributed to faster metabolism of olanzapine (43). However, the significance of these variants for the white population is still unknown.

The main role in glucuronidation of olanzapine is played by the enzymes uridine diphosphate glucuronosyltransferase, UGT1A4 and UGT2B10 (59). Our patient was homozygous for the variant UGT1A4*3, which implies accelerated metabolism (46) and increased glucuronidation of olanzapine (75), resulting in lower concentrations of the drug.

Olanzapine is also a substrate of P-gp/ABCB1. Some authors have reported on the association between *ABCB1 c.1236T>C* (rs1128503) T allele and elevated exposure to olanzapine, resulting in a better response to treatment (46,76). In another study, *ABCB1* rs3842 CC was associated with higher olanzapine exposure (61), while other gene variants were associated with variability in T1/2 (51). In our previous study in female schizophrenic patients, *MDR1/ABCB1 G2677T* carriers of the variant allele (T) had a better response to olanzapine (77).

We can conclude that in our patient, an accelerated metabolism due to *CYP3A*, *CYP1A2,* and *UGT* variants, together with the induction caused by carbamazepine, could have resulted in lower olanzapine concentrations.

### Carbamazepine

Carbamazepine is a strong inducer of multiple enzymes (CYPs and UGTs) and P-gp/ABCB1 transporter (78). Carbamazepine may interact with psychotropic drugs (79,80). Enzyme induction accelerates drug metabolism, while induction of P-gp changes the bioavailability of drugs at several levels: in enterocytes, the blood-brain barrier (BBB), and renal tubules. Induction enhances the function of the efflux pump, which results in a lower bioavailability of drug substrates (in this case olanzapine, risperidone, and aripiprazole). Induction of the ABCB1 transporter by carbamazepine at the gastrointestinal level means an increased efflux pump in enterocytes, induction at the BBB means a weaker passage of drugs into the brain, while induction in the kidneys means increased excretion of the drug.

The overall effect of induction with carbamazepine is reduced bioavailability of substrate drugs, and reduced entry into the brain, resulting in ineffectiveness. The AUC of aripiprazole (70%-90%) significantly decreases with the use of carbamazepine (81-83). In patients treated with 400 mg/day of carbamazepine, olanzapine exposure was lower by 36%-71% compared with patients on monotherapy (84-88).

Carbamazepine also has an effect on risperidone. A decrease in risperidone active moiety by 1.4 and 1.8 was reported with concomitant carbamazepine therapy (89,90). Risperidone active moiety doubled, resulting in ADR in a patient after carbamazepine discontinuation (91). On the other hand, a case study reported on the treatment for rapid reversal of long-acting risperidone by carbamazepine in a patient who developed extrapyramidal symptoms. The treatment relied on induction of CYP3A4 activity, since patients on long-acting antipsychotics need more time to excrete the offending drug (92). The gene variants of the enzymes CYP3A and UGT2B7 can also modulate the pharmacokinetics of carbamazepine (93). In our patient, the influence of enhanced metabolism via CYP3A and UGT2B7 was also observed. With 400 mg/day, a carbamazepine concentration of 10.4 μmol/L was achieved, while the expected range is 17-50 μmol/L. Physiologically based pharmacokinetic modeling is proposed to estimate the potential of carbamazepine interactions (94).

### Conclusions

We report on a complex case of a patient who had a pharmacogenetic predisposition for accelerated metabolism by several drug metabolizing enzymes (CYP3A5, CYP1A2, UGT1A4, UGT2B7). Concomitant therapy with carbamazepine as a strong enzyme inducer resulted in low concentrations of substrate drugs aripiprazole, risperidone, and olanzapine and an ineffective treatment.

The case shows that knowledge of pharmacogenetic predispositions along with careful evaluation of possible drug interactions can contribute to personalized and effective treatment of psychiatric patients. A multidisciplinary approach including a psychiatrist, clinical pharmacologist, and pharmacogeneticist is inevitable, especially today in the era of personalized and modern medicine.
